# Use of Temperature for Standardizing the Progression of *Francisella tularensis* in Mice

**DOI:** 10.1371/journal.pone.0045310

**Published:** 2012-09-24

**Authors:** Claudia R. Molins, Mark J. Delorey, John W. Young, Brook M. Yockey, John T. Belisle, Martin E. Schriefer, Jeannine M. Petersen

**Affiliations:** 1 Division of Vector-Borne Diseases, Centers for Disease Control and Prevention, Fort Collins, Colorado, United State of America; 2 Microbiology, Immunology, and Pathology Department, Colorado State University, Fort Collins, Colorado, United States of America; Albany Medical College, United States of America

## Abstract

The study of infectious agents, their pathogenesis, the host response and the evaluation of newly developed countermeasures often requires the use of a living system. Murine models are frequently used to undertake such investigations with the caveat that non-biased measurements to assess the progression of infection are underutilized. Instead, murine models predominantly rely on symptomology exhibited by the animal to evaluate the state of the animal's health and to determine when euthanasia should be performed. In this study, we used subcutaneous temperature as a non-subjective measurement to follow and compare infection in mice inoculated with *Francisella tularensis*, a Gram-negative pathogen that produces an acute and fatal illness in mice. A reproducible temperature pattern defined by three temperature phases (normal, febrile and hypothermic) was identified in all mice infected with *F. tularensis*, regardless of the infecting strain. More importantly and for the first time a non-subjective, ethical, and easily determined surrogate endpoint for death based on a temperature, termed drop point, was identified and validated with statistical models. In comparative survival curve analyses for *F. tularensis* strains with differing virulence, the drop point temperature yielded the same results as those obtained using observed time to death. Incorporation of temperature measurements to evaluate *F. tularensis* was standardized based on statistical models to provide a new level of robustness for comparative analyses in mice. These findings should be generally applicable to other pathogens that produce acute febrile disease in animal models and offers an important tool for understanding and following the infection process.

## Introduction

The ability to translate basic research findings for infectious diseases often relies on animal models to serve as surrogates for human illness. Mice are one of the most common animal models used to study infectious disease as they are susceptible to a similar range of microbial infections as humans. The field of infectious disease research lacks a robust substitute to animal models (e.g. living systems) for the study of pathogenesis, innate and adaptive immune responses, and efficacy of preventive or therapeutic interventions of disease. It is therefore critical that the study of infectious agents in mice and other animal models is performed in both an ethical and non-biased manner.

The use of non-subjective measurements to follow the course of disease in mice and that do not require a biological sample (e.g. heart rate, temperature, oxygen levels, weight) has not been well explored. Disease progression in mice is commonly assessed by symptoms such as piloerection, hunched posture, lack of eating and drinking, and slow response to stimulus; all of which are subjective indicators of disease state. Similarly, studies of fatal illness in murine models due to infectious diseases typically do not use death as an endpoint, and instead, animals are euthanized based on investigator interpretations of clinical symptoms or at specific intervals throughout the study. These approaches presume that individual animals are in the same state of infection when euthanized, thereby potentially introducing a bias or masking differences for survival analyses and other measurements, such as bacterial or viral burdens and immunological markers of disease. Given the central role of murine models to the study of infectious diseases, use of objective measures as surrogates of death and disease state is necessary to allow for standardization within and among analyses.

Several lines of evidence suggest that changes in temperature may serve as a non-biased indicator of infection status in mice [1]–[3]. Onset of a fever is a common symptom of many viral and bacterial infections. Hypothermia has been shown to correlate with toxicity and/or death in animals infected with a number of different pathogens including *Klebsiella pneumonia*, *Streptococcus pneumonia*, *Pseudomonas aeruginosa*, *Staphylococcus* spp., Influenza virus and *Candida* spp. [1]–[7], However, these previous studies did not employ statistical modeling to establish temperature profiles or defined measurements as determinants of disease progression or death.

In this study, subcutaneous body temperature was used to define and compare disease progression in mice intradermally infected with the bacterial pathogen, *Francisella tularensis. F. tularensis* is the etiologic agent of tularemia, with the onset of fever in humans being one hallmark of this disease [8]. This pathogen was chosen for the current work as it produces an acute fatal illness in mice. Moreover, virulence in mice has previously been shown to differ based on the infecting *F. tularensis* subspecies (*tularensis* and *holarctica*, also known as type A and type B, respectively) and subpopulation (A1a, A1b, and A2) [9] , thereby allowing for temperature to be explored in relation to ethical endpoints of survival. Temperature variances through the course of disease defined stages of infection and a single temperature measurement, termed drop point, was identified as an ethical, non-subjective and statistically valid correlate of death. The data also demonstrate that mice infected with the same *F. tularensis* strain did not progress through the infection simultaneously, thus underscoring the need for objective measurements of the infection process. The temperature model described here provides a new and robust tool for researchers using murine models to study the pathogenicity of bacteria that cause acute febrile infections, as well as for testing the efficacy of new drugs and vaccines developed against these agents.

## Results

### Phases of *F. tularensis* infection based on mouse subcutaneous temperature

**Figure 1:**
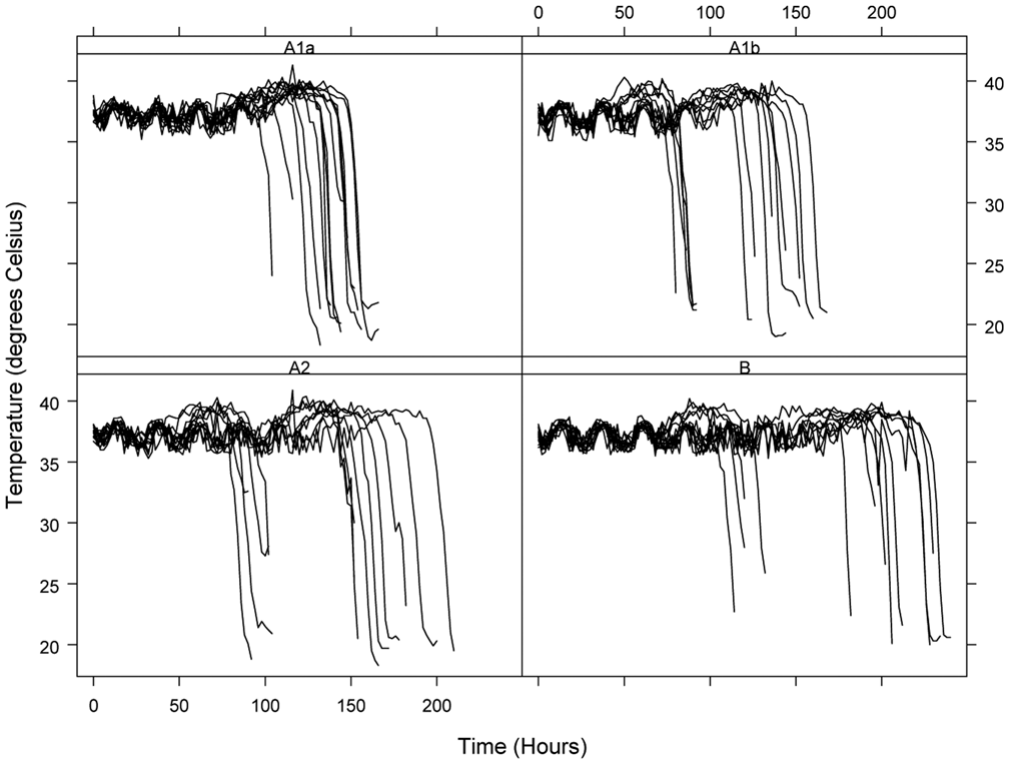
Temperature curves. Mice (n = 14 mice for each of the four *F. tularensis* infection groups) infected in round 1 and round 2 with *F. tularensis* groups (A1a, A1b, A2 and type B) are shown. Mice were infected with *F. tularensis* and temperature was monitored every 1–2 hours until mice expired.

**Figure 2:**
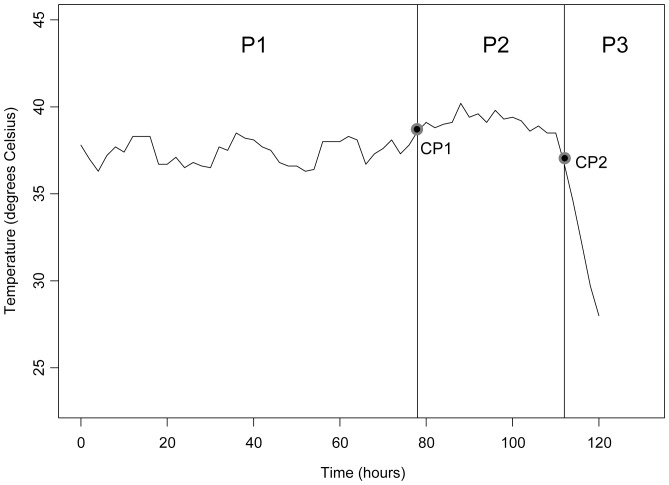
A representative mouse subcutaneous temperature curve in response to infection with *F. tularensis* type B is shown. P1, P2 and P3 represent phase 1 (normal phase), phase 2 (febrile phase) and phase 3 (hypothermic phase), respectively. Black dots on the temperature curve labeled CP1 and CP2 represent change point 1 and change point 2, respectively.

Temperature curves for mice infected with *F. tularensis* A1a, A1b, A2 and type B (n = 14 mice per *F. tularensis* group) were generated and analyzed (Figure 1). Diagnostic plots created from the 56 individual temperature curves suggested that the time series is autoregressive with dependencies at lags of 1 time period (2 hours) and 12 time periods (24 hours). The lag at 2 hours indicates successive temperature measurements are correlated while the lag at 24 hours indicates temperature measurements taken at the same time each day are correlated. The method of Barry and Hartigan [10] identified two change points (a time point at which some fundamental characteristic such as the mean process or variance structure of the time series changes) in the temperature time series for all 56 *F. tularensis* infected mice. A representative temperature curve with the two change points, (CP1 and CP2), and intervening phases (P1, P2 and P3) is shown in Figure 2.

Comparison of mouse subcutaneous temperature in P1 indicated that the subcutaneous temperature of *F. tularensis* infected mice fell within the tolerance interval (35.3°C to 38.6°C) nearly 99% of the time. Temperature fluctuation during this phase ranged from 35°C to 39°C and was consistent with the temperature of uninfected animals. Mice were asymptomatic throughout this phase, therefore, P1 was termed the “normal phase”. The second phase (P2) of *F. tularensis* infection began with a rise in temperature that was sustained over the time course of this phase. In P2 the subcutaneous temperature of *F. tularensis* infected mice fell within the tolerance limits only 33% of the time. Mice showed slight clinical symptoms, primarily huddling within their enclosure, but remained responsive and continued to eat and drink throughout most of this phase. Due to the elevated temperature (>38°C) maintained by infected mice during this period, P2 was termed the “febrile phase”. The last temperature phase of *F. tularensis* infection consisted of a sudden drop from elevated temperatures with temperatures continuing to drop until death of the mouse. Thus, P3 was termed the ‘hypothermic phase”.

In addition to the three phases of *F. tularensis* infection demarcated by temperature CP1 and CP2, other similarities were noted among the temperature responses in mice infected with *F. tularensis*. Autocorrelation plots indicated strong evidence of a diurnal effect on temperature during the normal phase of *F. tularensis* infection. The temperature fluctuation during this phase (35°C to 39°C) correlated directly to mouse activity over a 24 h period. The daily temperature cycle was much less pronounced in the febrile phase, although this could be because the length of the febrile phase was generally much shorter (mean length of 38 to 42 h) than the normal phase (mean length ranging from 77 to 138 h) (see Table 1), but is more likely due to the decrease in mouse activity attributable to *F. tularensis* infection. The hypothermic phase averaged 10 to 15 h and the mice did not present daily cyclical behavior.

**Table 1:** Length of infection phase for mice infected with each *F. tularensis* group (A1a, A1b, A2 and type B).

Phase	Length Measured^a^	*F. tularensis* group			
		A1a	A1b	A2	type B
Normal	Mean length	91.3	76.5	97.1	138.4
	Range of length	(60.0, 108.0)	(34.0, 114.0)	(36.0, 160.0)	(76.0, 186.0)
	1st percentile distribution	56.60	21.90	22.30	53.70
	5th percentile distribution	68.50	35.60	39.20	77.80
	10th percentile distribution	74.50	44.00	50.20	91.6
Febrile	Mean length	40.30	39.10	37.70	42.40
	Range of length	(28.0, 50.0)	(28.0, 56.0)	(30.0, 50.0)	(28.0, 62.0)

a All lengths were measured in hours.

The temperature pattern of infection was similar for all mice, as all mice underwent P1 to P3 (Figure 1). The mean length of each phase differed based on the infection group (*F. tularensis* A1a, A1b, A2 or type B) (Table 1). It was also observed that mice infected with a single strain did not succumb to infection or reach a particular phase or change point at the same time (Figure 1, Table 1). Mice infected with different strains of the same group were only in the same phase for 33% (2 out of 6 days), 14.3% (1 out of 7 days), 12.5% (1 out of 8 days) and 30% (3 out of 10 days) of the time for A1a, A1b, A2 and type B, respectively (Table 2).

**Table 2:** Number of mice in each phase over time.

*F. tularensis* infection group^a^	Phase	Time (hours)										
		24	48	72	96	120	144	168	192	216	240	264
A1a	Normal	14	14	**13** b	**7**	0	0	0	0	0	0	0
	Febrile	0	0	**1**	**7**	**11**	**2**	0	0	0	0	0
	Hypothermic	0	0	0	0	**1**	**6**	0	0	0	0	0
	Expired	0	0	0	0	**2**	**6**	14	14	14	14	14
A1b	Normal	14	**11**	**9**	**4**	0	0	0	0	0	0	0
	Febrile	0	**3**	**4**	**5**	**7**	**3**	0	0	0	0	0
	Hypothermic	0	0	**1**	0	**2**	**3**	**1**	0	0	0	0
	Expired	0	0	0	**5**	**5**	**8**	**13**	14	14	14	14
A2	Normal	14	**12**	**9**	**9**	**5**	**1**	0	0	0	0	0
	Febrile	0	**2**	**5**	0	**4**	**6**	**3**	**1**	0	0	0
	Hypothermic	0	0	0	**3**	0	**2**	**2**	**1**	0	0	0
	Expired	0	0	0	**2**	**5**	**5**	**9**	**12**	14	14	14
type B	Normal	14	14	14	**10**	**10**	**9**	**4**	0	0	0	0
	Febrile	0	0	0	**4**	**1**	**1**	**6**	**8**	**4**	0	0
	Hypothermic	0	0	0	0	**2**	0	0	**1**	**1**	**1**	0
	Expired	0	0	0	0	**1**	**4**	**4**	**5**	**9**	**13**	14

a Infection data from rounds 1 and 2 were combined.

b Bold and underlined numbers represent mice that are not in the same phase at a particular time.

### Time to death predictions based on temperature change

**Figure 3:**
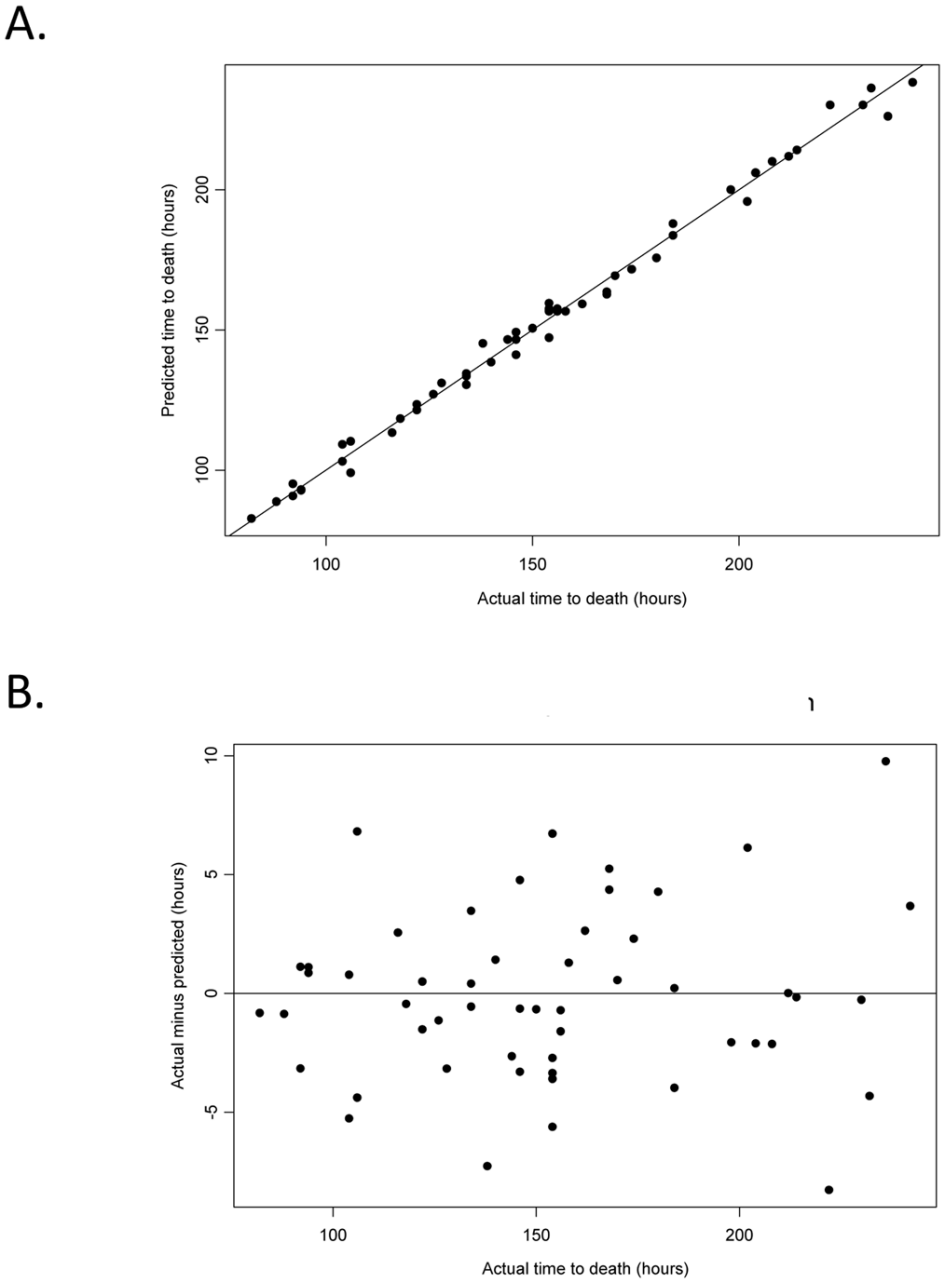
Predicted time of death (based on change point 2) versus observed time of death. A) The correlation between predicted time of death and observed time of death are shown for all mice (black dots) infected with *F. tularensis* groups (A1a, A1b, A2 and type B) in both round 1 and round 2. The solid black line represents 100% correlation. B) The observed time of death minus the predicted times of death are shown for all mice (black dots) infected with *F. tularensis* groups (A1a, A1b, A2 and type B) in both round 1 and round 2.

To determine whether temperature data serves as a correlate to death, the mean temperature during the normal phase, the mean temperature during the febrile phase, CP1, or CP2, for *F. tularensis* group (A1a, A1b, A2 and type B) were used in the linear mixed-effect model to predict time to death. The mean temperature during the normal and febrile phase were found to be uncorrelated with time to death, whereas CP1 and CP2 (Figure 3A) both correlated with the observed time to death. CP2 was pursued further as a time to death predictor as it occurred later in the progression of infection and therefore provided information for the infection through P2. The differences (in hours) between the observed and predicted times of death based on CP2 are shown in Figure 3B. The maximum error of prediction as compared to observed time to death is about 10 h with 71% of the predictions being within 5 h of the observed time to death. The square root of the mean square prediction error (RMSPE) for a leave-one-out validation is 3.89 h; for a leave-five-out validation, it is 3.92 h; and when half of the data set is left out (leave-28-out), the RMSPE is 4.09 h. Compared to the RMSPE for the full set of data (leave-zero-out) of 3.58 h, these suggest a strong predictive value of CP2 for time to death.

### Identification and definition of drop point for use as a surrogate endpoint

As determination of CP2 required complex statistical analyses, temperature curves where further analyzed to elucidate an easily identifiable point in the infection that could be readily applied as a surrogate for time to death. With only two exceptions (n = 56 mice) that were due to misreads by the probe, CP2 was found to be the first temperature measurement following the febrile phase to fall below the mean temperature of the normal phase (Figure 2). Therefore, this temperature change was termed “drop point”. The drop point differed slightly for each mouse given that it is directly dependent on the mean of the normal phase. Further investigation of the length and frequency of temperature monitoring necessary to determine drop point based solely on the mean temperature of the normal phase was performed. Calculating the 1^st^ percentile of the distribution of the length of P1 (mean length of time for P1 was 91.3, 76.5, 97.1 and 138.4 h in A1a, A1b, A2 and type B infected mice, respectively) indicated that the temperature of mice infected should be monitored for at least 56.6 h for A1a, 21.9 h for A1b, 22.3 h for A2, and 53.7 h for type B in order to accurately identify the drop point (Table 1). To determine the frequency of temperature monitoring necessary, the percent agreement between CP2 (based on joint likelihood analysis of time series plots) and drop point (the first temperature below the mean temperature of the normal phase) was computed for 5000 of temperature data for each of the 56 mice infected with *F. tularensis*. When temperature was sampled every 2 h, CP2 and drop point analyses identified the same time point 96% of the time. When temperature was sampled at 4, 6 and 12 h intervals, the agreements were 95.4%, 94.9%, and 92.1%, respectively. These results suggest that monitoring frequency should be no less than every 6 h to appropriately identify the drop point.

### Comparative virulence based on predicted vs. observed time to death

**Figure 4:**
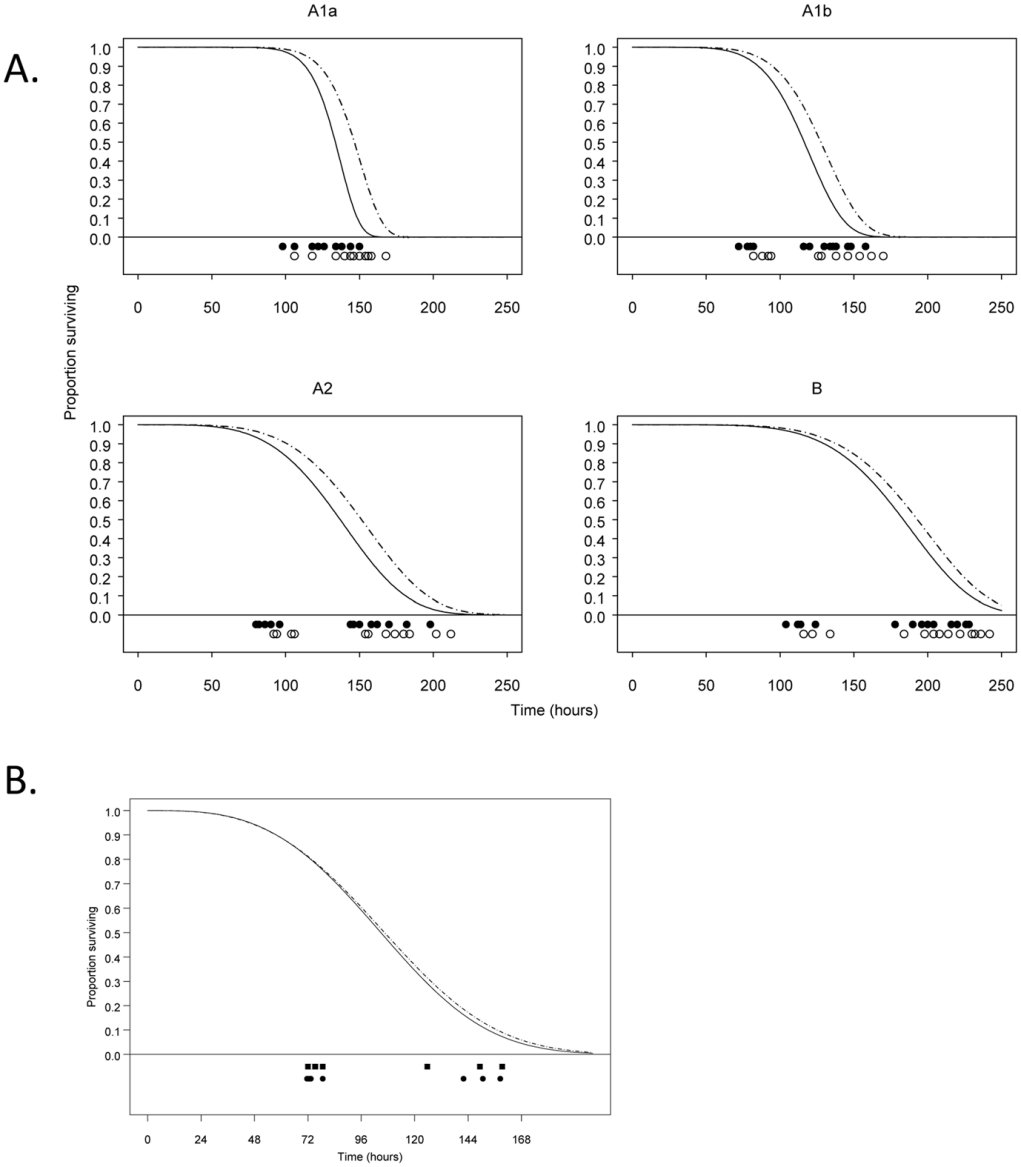
Comparison of fitted survival curves. A) Survival curves (fitted for all data from round 1 and round 2) are shown for mice infected with *F. tularensis* groups (A1a, A1b, A2 and type B) using drop point (solid lines) and observed time of death (dashed lines). The time at which each mouse expired (solid circles) or reached drop point (open circles) was reached is shown below the graph. B) Survival curve comparison between two infections performed at different times using the same A1b strain MA00-2987. The first MA00-2987 infection is denoted as (••• — •••). During this infection, mice were allowed to expire and drop point was subsequently identified and used as the endpoint for generating the survival curve. The second MA00-2987 infection is denoted as (——). Mice were euthanized at drop point during this infection for generation of the survival curve. The time at which each mouse reached drop point is shown below the graph as squares (1^st^ round) or circles (2^nd^ round).

We previously showed that the parametric survival curves (modeled according to a Weibull distribution) for mice infected with *F. tularensis* A1a, A1b, A2 or type B are statistically different from one another in scale (i.e. mean and variability of time to death) and/or shape (i.e. symmetry of pattern of death) using observed time to death as the endpoint [9]. Therefore we investigated whether survival curves based on predicted time to death using CP2 or drop point yielded statistically similar results when compared with observed time to death. The differences observed among *F. tularensis* infection groups were the same as those obtained from observed time to death experiments when parametric survival curves were generated based on either CP2 (data not shown) or drop point (Figure 4A). Specifically, the scale parameter of survival curves differed significantly (p<0.008) between A1b infected mice and those of A1a, A2 and type B infected mice as well as between A1a and A2 infected mice. The survival curve for mice infected with A1a differed significantly (p<0.008) in shape from the survival curves of mice infected with A1b, A2 and type B. Although the prototypic *F. tularensis* virulent strain Schu S4 was not included in this study, it should be noted that this strain is classified as an A1a strain [11]–[12] Survival curves based on CP2 were statistically the same as the survival curves based on drop point (data not shown). These results indicate that predicted time to death based on CP2 or drop point can readily substitute as a non-biased surrogate endpoint for time to death experiments.

### Experimental determination and utilization of drop point for survival curves

To further assess the utility of using drop point as an ethical endpoint for survival curve analysis, mice (n = 7) were infected with *F. tularensis* A1b strain MA00-2987, the same strain used in previous observed time to death experiments [9], and mouse temperature was monitored every two hours over the mean length of the normal phase for A1b (21.9 hours). The mean temperature of the normal phase was determined for each mouse and mice were euthanized once the drop point temperature was observed. A survival curve using drop point as the endpoint was generated. This was then compared to a *F. tularensis* MA00-2987 survival curve using drop point data calculated from a previous observed time to death experiment [9]. No significant differences (p = 0.89 for scale parameter; p = 0.95 for shape parameter; log-likelihood ratio p = 0.99) were observed in these two survival curves (Figure 4B), thus demonstrating the accuracy and robustness across experiments as well as the feasibility of using the drop point temperature as a surrogate endpoint for death.

## Discussion

Animal studies provide essential information in the evaluation of novel drugs and vaccines for human infectious diseases, and the robustness of the model impacts the quality and utility of the resulting data. Elevated temperature is a phenotype common to many infectious diseases and is noted as a correlate of pathogenesis [13]. The current study applied statistical modeling to advance the use of temperature measurements such that a surrogate marker of death for *F. tularensis* infection in mice was defined. Two surrogate endpoints for death based on temperature were identified. The first of these termed CP2 occurred consistently between the febrile (P2) and hypothermic phases (P3) of disease, but could only be defined by linear mixed-effect modeling. Thus, CP2 could not be determined in real-time during the course of an experiment and could not be applied as a surrogate of death. Inspection of temperature curves, however, indicated that CP2 closely correlated to the first temperature measurement at the end of the febrile phase (P2) falling below the mean temperature of the normal phase (P1), and thus was termed “drop point”. It was noted that mice at the drop point were responsive to stimuli and still taking food and water. Independent survival curves generated for *F. tularensis* strains with varying virulence and based on CP2 or drop point were statistically indistinguishable from those obtained using observed time to death. The experimental feasibility of determining and using drop point as an accurate indicator of death was also shown through survival curve comparisons that showed no difference between mice infected with *F. tularensis* Alb strain MA00-2987 euthanized at drop point and mice previously infected with the same strain for which drop point was calculated after they were allowed to expire. Together these results demonstrate that the easily determined drop point can be applied as an ethical, non-biased and accurate surrogate endpoint for death in mice infected with *F. tularensis*. Given that fever is a characteristic symptom of tularemia [8], this approach can be further developed and modified for use in other animal models, and for different inoculation routes and doses. Further, the application of statistical modeling to temperature profiling should be applicable to the study of other acute infectious agents that cause febrile illness.

The data generated through our studies not only demonstrated that the drop point was a statistically valid surrogate endpoint for death, but also revealed that temperature profiling can be used to follow the variability in disease progression between infected animals to a level not previously accomplished. A previous assessment of the virulence of eight diverse *F. tularensis* strains in the C57BL/6 mouse model based on time to death reveled that animals succumbed to infection between 82 and 242 h and the variance in time to death for a single strain was between 51 and 129 h [9]. The evaluation of body temperature of C57BL/6 mice intradermally infected with the same eight strains of *F. tularensis* revealed three temperature phases (normal, febrile and hypothermic) demarcated by two change points (CP1 and CP2) that occurred regardless of the strain. However, as was observed in the variance of time to death [9], mice advanced through the stages of infection as defined by temperature at dramatically different rates. This variability was not only between strains, but there was a notable variance for animals infected with the same *F. tularensis* group, with less than 34% congruency with respect to the number of days in which mice were within the same temperature phase. Such variation among mice infected with *F. tularensis* is not unique to this study [14]. In fact, previous studies show that bacterial burdens analyzed for *F. tularensis* infected mice that were euthanized based on a given day post infection differed by as much as 7 Log_10_ CFU/organ [14]–[17]. Differences in disease progression as indicated by our subcutaneous temperature profiles would be consistent with variance in other measures of disease progression. Further, the majority of animal studies performed with *F. tularensis* collect and test biological samples at specific intervals (24, 48, 72 h, etc.) [14]–[16], [18]. Thus, with an acute disease and one that is significantly influenced by the route of infection and minor variations in the infecting inoculums, the practice of collecting biological samples at specific time intervals rather than at defined points of disease likely contributes to the variability in the biological analyses of disease. Temperature monitoring therefore, provides a reproducible picture of infection progression; a feature critical for comparative animal studies within and between laboratories, and one that could be applied to normalize sample collection or biological data.

A non-biased temperature measurement of infection in animal models is advantageous in that it is minimally invasive, requiring the subcutaneous implantation of a probe via injection. A biological sample is not required from the animal and measurements are easily taken without disrupting or causing stress to the animals. Such a system has value in guiding the experimental administration of therapeutics as well as sample collection. As demonstrated by Bast et al. temperature provides a measure of pneumococcal pneumonia severity in mice, and when used in the evaluation of moxifloxacin and levofloxacin efficacy this group demonstrated that animals receiving antibiotics at temperatures ≥32°C fared better than those receiving antibiotics at temperatures of <30°C [19]. When combined with appropriate statistical modeling, telemetry based measurements that include other physiological parameters such as blood pressure, biopotential (ECG, EEG, and EMG), heart rate and temperature offer an extraordinary tool to maximize animal resources and normalize experimental data for acute infectious diseases such as that caused by *F. tularensis*. Additionally, as telemetry technology advances, automation of this system for use in mice will allow drop point and other measurements identified to be readily used as surrogate endpoints for death.

## Materials and Methods

### Ethics Statement

All animal procedures were approved by the Division of Vector-Borne Diseases Institutional Animal Care and Use Committee (protocol number 08-012) and performed in accordance with the guidelines on the care and use of laboratory animals [20].

### Mice and experimental protocol

Specific-pathogen free 8–9 week old female inbred C57BL/6 mice (The Jackson Labortory, Bar harbor, ME) were used. Mice were implanted a week prior to *F. tularensis* infection with IPTT-300 transponders (BioMedic Data Systems Inc., Seaford, DE) programmed with mouse identifiers. Mice were anesthetized by inhalation with isoflurane and transponders were implanted below the dermis in the upper back region as per the manufacturer's instructions. Mouse subcutaneous temperature was monitored using the DAS-6007 probe (BioMedic Data Systems Inc.) once a day prior to infection and every 1 to 2 hours following infection until the mice expired. Control mice injected with saline were used to monitor normal mouse subcutaneous temperature throughout the study. Mice were given food and water ad libitum, and an exercise wheel was provided in every cage. Infection of mice with strains representing each of the four *F. tularensis* groups (A1a, A1b, A2 and type B) was accomplished by inoculation of 10–20 CFU intradermally (50 µl) via the tail dermis and colony forming units in each inoculum were verified as previously described [9]. For temperature model development, mice were allowed to expire. Mice (n = 7 in each group) were infected in 2 rounds. The first round consisted of infection with one strain representing each of the four *F. tularensis* groups (A1a, A1b, A2 and type B). The second round was a repeat of the first round using a different set of strains. A final animal infection was performed to test the temperature model. In this case mice were infected with *F. tularensis* strain MA00-2987 (n = 7) as described and temperature was monitored every two hours. The mean temperature of the normal phase was calculated once mice entered the febrile phase and mice were euthanized at their drop point. Two criteria needed to be satisfied to conclude a mouse had entered the febrile phase: first, the length of time passed must be greater than the 5^th^ percentile of the distribution of the length of the normal phase, and second, the temperature must remain above the cumulative mean temperature for 12 consecutive hours. All animal experiments were conducted in an ASBL3 facility.

### Strains and culture conditions

*F. tularensis* strains (n = 8) used in this study originated from human tularemia cases [9]. Strains were grown from frozen stocks (−70°C) on cysteine heart agar supplemented with 9% sheep blood (CHAB) at 35°C for 48 h, followed by subculture onto CHAB for 24 h at 35°C. All *F. tularensis* strains were typed using pulsed-field gel electrophoresis (PFGE) as previously described [11], [21].

### Subcutaneous temperature model of *F. tularensis* infection

Time series plots of temperatures were generated using Spotfire S+ v8.1 (TIBCO Software, Inc.). Autocorrelation and partial autocorrelation plots were created for different contiguous subsets of the data in order to determine approximate structure of the moving average and autocorrelation components of the time series. To identify the number of change points (different segments of the time series), the method described by Barry and Hartigan [10] was implemented. To identify the location of the change points, the joint likelihood of the time series segments was constructed. Temperatures were assumed to be statistically independent among the segments. The mean process in all but the final segment was assumed constant (but different). The mean process in the final segment was modeled by a second degree polynomial function of time. The variances of the segments were allowed to differ by segment, but were assumed constant within each segment. The joint likelihood of the data was maximized over a grid of change point values.

For temperature comparisons between control and infected mice the marginal mean and variance of the distribution of temperatures in the control mice were estimated using a linear mixed-effects model accounting for mouse effects and AR1 correlation among temperatures within mice. Diagnostics, including quantile-quantile plots were used to verify the assumption of normality. Using the marginal mean and variance, a 95% tolerance interval for temperatures in the control mice was constructed. By construction, a temperature sequence consistent with that found in the control mice should fall within the tolerance limits 95% of the time. This tolerance interval was mapped onto the temperature sequences of the infected mice to determine if normal phase temperatures and febrile phase temperatures in infected mice were consistent with temperatures in control mice.

### Time to death prediction

Using a linear mixed-effect model, time to death was modeled as a function of temperature data (time of change points, mean temperature during normal phase, and mean temperature during febrile phase). *F. tularensis* group (A1a, A1b, A2 and type B) was included as a random effect. Standard diagnostics, including residual analysis, were employed to ensure a valid model. The model was fit using data from all infections, and a cross-validation on the model was performed leaving out subsets of size one to 50% of the data and calculating the RMSPE (an estimate of the average number of hours by which the predicted times of death differed from the observed times of death).

### Duration and frequency of monitoring times needed to accurately estimate the mean of the normal phase

Distribution plots of the lengths of the normal phase were made and Weibull densities were fit to these lengths for each *F. tularensis* group. The 1st, 5th, and 10th percentiles for each distribution were then estimated. Using the estimated models from the original data [9] we simulated 5000 new sets of temperature data for each mouse. Under sampling frequencies of 2, 4, 6, and 12 h and sampling to the 5^th^ percentile, the proportion of times among all simulations in which the sample mean temperature during the normal phase was higher than the sample minimum temperature during the febrile phase was computed.

### Comparative virulence

As described previously [9], survival times were modeled according to a Weibull distribution (allowing both the scale and shape parameters to differ) using predicted time to death and drop point as endpoints. Standard diagnostics including residual plots and goodness of fit tests were used to validate the model fits. The survival curves generated here were compared to those created using observed time to death [9]. Differences in the parameter estimates (shape and scale) of the survival curves were considered statistically significant if p<0.008, with this level of significance determined using the Bonferroni adjustment for multiple comparisons.
